# Enhancement of microbiome management by machine learning for biological wastewater treatment

**DOI:** 10.1111/1751-7915.13707

**Published:** 2020-11-22

**Authors:** Wenfang Cai, Fei Long, Yunhai Wang, Hong Liu, Kun Guo

**Affiliations:** ^1^ School of Chemical Engineering and Technology Xi'an Jiaotong University Xi'an 710049 China; ^2^ Department of Environmental Science and Engineering Xi'an Jiaotong University Xi'an 710049 China; ^3^ Department of Biological and Ecological Engineering Oregon State University Corvallis OR 97331 USA

## Abstract

Here, we propose to develop microbiome‐based machine learning models to predict the response of biological wastewater treatment systems to environmental or operational disturbances or to design specific microbiomes to achieve a desired system function. These machine learning models can be used to enhance the stability of microbiome‐based biological systems and warn against the failure of these systems.

## Abstract 2

Here we propose to develop microbiome‐based machine learning models to predict the response of biological wastewater treatment systems to environmental or operational disturbances or to design specific microbiomes to achieve a desired system function. These machine learning models can be used to enhance the stability of microbiome‐based biological systems and warn against the failure of these systems.

Microbiomes play the key role in biological wastewater treatment processes such as activated sludge, anaerobic digestion and bioelectrochemical systems. The performance and stability of these processes are highly depending on the activity and stability of the microbial community in the microbiome. However, the complexity of the microbiome (i.e., types of microbes, the function of each microbe and the interactions among different microbes) makes it difficult to precisely control these wastewater treatment processes and hard to predict the performance of them. Consequently, frequent maintenance is required to these processes, which in return results in low efficiency, high energy input and poor performance of the systems. Thus, successfully managing the microbiomes in order to improve their stability and activity in these biological wastewater treatment processes can lead to higher efficiency, lower energy input and more products.

Currently, there are three different approaches to manage microbiomes, namely retrospective management, prospective management and proactive management (Carballa, *et al*., 2015). Retrospective microbiome management simply recorded system performance changes (success or failure) first and then explain the changes by microbiomes shift. Retrospective management is effective, but the conclusions might be unreliable for the following reasons: (i) the performance fluctuation may be caused by several simultaneous disturbances; (ii) the limitations of understanding the highly complex relationship between system performance and microbiomes; (iii) the lack of comprehensive monitoring all parameters of system dynamics. Prospective management designs a perturbation experiment with detailed monitoring each operated parameter and performance indicator, and then, the relationship between microbial community structures and the system functional changes could be established. In this type of management, one waits for process failure and then finds a solution to remediate it. On the contrary, proactive management focuses on identifying and assessing all potential disturbances and developing strategies to prevent process failure. Thus, proactive microbiome management has a great potential for preventing process failure (Stenuit and Agathos, 2015). This kind of microbiome management needs early responded microbial indicators (i.e., some microbial communities changed before macroscopic performance reacted to disturbances) to provide early guidance for instability warning and guaranteeing system function. The biggest problem for developing this kind of microbiome management is the huge experimental works needed for routine microbiomes analysis and system performance monitoring. Even though it is possible to convert DNA sequencing data to meaningful microbial patterns and microbial related datasets, we could only obtain limited information about the relationship between microbiomes dynamics and system function robustness from experiments. More importantly, proactive microbial management from experiments is based on the hypotheses that early responded microbial indicators have been set down. All mentioned above hinder the development of proactive microbiome management to maintain the system performance stability, strategies need to be adopted to proactively manage microbiome dynamics to prevent process failure.

Thanks to the rapid development of microbiome diagnostics (Koch, *et al*., 2014), analysis of reactor microbiomes become more and more feasible, and large amount of microbial information data could be obtained. Models could be developed to warn and prevent system function failure with the microbial information. However, exploring models to predict system’s functions via incorporating the dynamics of microbial communities is still in its infancy. The universal stochastic/metabolic models are not feasible for obtaining temporal and spatial dynamics of microbial communities (Bucci and Xavier, 2014). The dynamic (the presence, absence and changes in abundance) of early responded microbes and the specific functional microbes could be used as indicators of process fluctuation caused by environmental stress. If the complex, multivariate and non‐linear relations between the dynamics of microbiomes and system performance could be elucidated, people could predict system performance based on the microbial indicators and thereby preventing system from failure. Machine learning has the potential to identify these more complex relationships through analysis of large datasets (Jiang and Hu, 2016). Moreover, machine learning models could also carry out high‐throughput modelling from massive microbial datasets for mining hidden information that could not be obtained via statistical methods. Several supervised machine learning algorithms have been adopted to predict feed substrates in microbial fuel cells by incorporating genomic data and thus increasing chemical detection specificity (Cai, *et al*., 2019). Besides, machine learning algorithms have also been used in anaerobic digestion process to identify determining operation parameters for biogas production (Wang, *et al*., 2020). More interestingly, artificial neural network, a kind of machine learning approach, has been applied in microbial fuel cells to predict microbiome response and reactor performance over a wide range of conditions (Lesnik and Liu, 2017).

Machine learning also has the potential to be a tool for proactive microbiome management since it could predict the system performance fluctuation caused by environmental stress using microbiome dynamics and operation parameters as input datasets. And it could also be used to identify the early responded microbial indicators by extracting the relative coefficient between microbiomes and performance fluctuation. Algorithms have been developed to directly predict system function from given microbiome structures, for example inferring reactor performance from pH disturbance (Lesnik, *et al*., 2020). This prediction was based on microbiome structure at a stable state after the disturbing, while microbiomes dynamics also play important role in system function stability, especially the early responded microbial indicators dynamics. Machine learning could also be used to design microbiomes to achieve a certain desired system function in reverse. This will make it possible to design microbiome diversity, relative abundance and the interactions needed to fulfil the promise of specific engineering goals (Lawson, *et al*., 2019).

Selection of suitable algorithms for performance prediction or specific microbiomes design is very important. Recursive neural networks (RNN) and convolutional neural networks (CNN) have shown great ability in modelling and forecasting non‐linear and non‐stationary time series (Mishra and Desai, 2006; Ince, *et al*., 2016). This kind of algorithms can be used to connect microbiome dynamics with performance changes, which is beneficial to warn reactor performance fluctuation from early responded microbial indicators to set edge cases to define the normal scope of operation. They could also identify the most sensitive parameters and unknown microbial indicators in reverse. Experimental exploration could be used to verify new hypotheses derived from machine learning models under designed operation conditions. Random forest (RF), support vector machine (SVM), partial least‐squares (PLS), neural networks (NNET), XGBOOST, etc. have always been used in biological wastewater treatments and are beneficial for some classification and regression problems (Cai, *et al*., 2019; Lesnik, *et al*., 2020). When these algorithms are being incorporating with microbiomes data in biological wastewater treatments, and the maximum accuracy of system function prediction reached to 93%, while the predictability of some algorithms was lower than 50% (Lesnik and Liu, 2017; Cai, *et al*., 2019; Lesnik, *et al*., 2020). The predictability of machine learning algorithms mentioned above is highly dependent on the size of datasets. Alternatively, rapid development of deep learning neural network (DLNN) has great potential to effectively and flexibly mine highly varying non‐linear functions based on multiple non‐linear hidden layers, such as the deep belief networks (DBN) and stacked auto‐encoders (SAE) networks (Lu, *et al*., 2015). The SAE network algorithm contains unsupervised pre‐training phase and the supervised fine‐tuning phase to maximizing prediction accuracy. Especially, stacked denoising auto‐encoders (SDAE) deep learning network derived from SAE network could get deep feature and accurate predictive results from limited experimental datasets containing microbiome information due to its anti‐interference ability and feature extraction ability (Vincent, *et al*., 2008).

Dataset is another core component in the predictability of machine learning models. The success of machine learning prediction is dependent on the quality of the datasets. In biological wastewater treatment process, microbiomes’ metabolisms could be affected by many factors, such as react configurations, operation and environmental conditions. Even though many abiotic and biotic measurement sets are applied, large datasets are often not available for a specific system function goal. For example, pH, temperature, substrate loading and inoculum all could affect the microbiome dynamics, but only one experimental variable can be studied at a time to verify relations between system function and microbiome. Besides, the impact of some parameters on the function stability may be not the determining factors. RF variable importance measures could be used to determine the impact of each variable of datasets as well as the multivariate interactions with other variables (Cai, *et al*., 2019). For microbiomes datasets, the presence or absence of the specific functional microbes, the microbiomes interactions and the microbiome dynamics have different impact on different system functional requirements. Functional microbes are indispensable for achieving specific functional stability under specific operation conditions. For example, in anaerobic digestion process, the presence of *Clostridia* class could be beneficial for degrading both protein and cellulose (Carballa, *et al*., 2015), while the presence of *Syntrophomonas* and *Synergistetes* might be a sign of good acetogenic and acetotrophic performance (Nelson, *et al*., 2011; Regueiro, *et al*., 2014). In bioelectrochemical systems, *Geobacter* and *Shewanella* are the most commonly identified exoelectrogens for current production, while methanogens result in electron sink at the anode (Borole, *et al*., 2011; Logan, *et al*., 2019). In aerobic denitrification process, *Hyphomicrobium* and *Methylotenera* represent obligate aerobes for utilizing both O_2_ and NO_3_^‐^ or NO_2_^‐^ as terminal electron acceptors, while *Pseudomonas* prefers using NO_3_^‐^ than O_2_ in aerobic denitrifier (Zhu, *et al*., 2016). However, datasets only containing these functional microbes are not enough for a successful machine learning model for microbiome‐based biotechnologies. Microbial interactions could also be used as machine learning input datasets to predict functional performance fluctuation caused by environmental or process disturbances. Similarity‐based network inference or regression‐ and rule‐based networks (Faust and Raes, 2012; Stenuit and Agathos, 2015) have been built to visualized microbiome interactions under the same operation conditions or different conditions and then being converted to numerical data, such as relative coefficient as machine learning input datasets. Most importantly, the microbiome dynamics over time is crucial for maintaining system functional stability and robustness. It could be adjusted according to the operational or environmental disturbance, providing system with access to functional specificity and flexibility. It is significantly important to indicate system performance fluctuations under disturbances from cross‐sectional at one time point to time series based on microbiome dynamics. Microbiome dynamics, especial early responded microbial indicators dynamics, being combined with operation conditions could be used as input datasets for warning against performance failure. For specific functional microbiomes design, relative coefficient extracted from interactions between system function and microbiomes and desired system performance indicators could be used as input datasets to predict microbiome compositions.

In summary, achieving microbiome management using machine learning models will have an important impact in biological wastewater treatment development, by shortening experimental research period and minimizing experimental work. Suitable machine learning models with specific input datasets (incorporating the information of microbiomes along with environmental conditions and operation parameters) provide a new approach for better managing microbiomes and eventually promote biological wastewater treatment operation. Model accuracy could be further improved with new experimental results. Therefore, combining experimental study with machine learning will be more efficient and effective on enhancing the performance of biological wastewater treatment systems.
